# A Functional Screen for Regulators of CKDN2A Reveals MEOX2 as a Transcriptional Activator of INK4a

**DOI:** 10.1371/journal.pone.0005067

**Published:** 2009-04-02

**Authors:** Jeffrey T. Irelan, Ana Gutierrez del Arroyo, Abel Gutierrez, Gordon Peters, Kim C. Quon, Loren Miraglia, Sumit K. Chanda

**Affiliations:** 1 Genomics Institute of the Novartis Research Foundation, San Diego, California, United States of America; 2 CRUK London Research Institute, London, United Kingdom; Ordway Research Institute, United States of America

## Abstract

The CDKN2A locus encodes two important tumor suppressors, INK4a and ARF, which respond to oncogenic stresses by inducing cellular senescence. We conducted a genome-scale cDNA overexpression screen using a reporter containing INK4a regulatory sequences to identify novel transcriptional activators of this locus. This screen revealed 285 cDNAs that putatively regulate the transcriptional activation of INK4a. Of these, 56 are annotated as transcription factors, including two previously reported activators of the locus, ETS2 and JUNB. Fourteen genes were further validated for activity and specificity, including several homeodomain proteins. We found that the transcription of one of these, the homeodomain protein MEOX2 (GAX) is enhanced in primary cells during the induction of senescence, and forced expression of this protein results in the induction of premature senescence. We further demonstrate that MEOX2-induced senescence is dependent upon INK4a activity, and chromatin immunoprecipitation studies indicate that MEOX2 directly binds the INK4a promoter. These results support a role for this homeodomain protein as a direct regulator of INK4a transcription and senescence in human cells.

## Introduction

The human CDKN2A locus encodes two important tumor suppressors, p16INK4a and p14ARF. This locus was initially discovered due to its linkage with a familial melanoma susceptibility syndrome, and subsequent work has demonstrated frequent loss of functionality of one or both of these genes in a wide variety of sporadic tumors (reviewed in [1]). Mouse models which harbor deletions in either or both genes reveal that, although neither is essential for embryonic development, both are involved in suppressing various tumor types (reviewed in [2]). INK4a and ARF are completely nonhomologous proteins, with distinct biochemical functions, yet the transcription of both genes increases in response to similar signals, resulting in an exit from the cell cycle (reviewed in [3]). Transcription of INK4a and ARF is driven by unrelated promoter sequences located 13 kb apart; distinct first exons for each gene are spliced to shared second and third exons that are translated in different open reading frames. The resulting INK4a gene product is a direct inhibitor of cyclin-dependent kinases 4 and 6. By preventing their interactions with D-type cyclins, INK4a prevents CDK 4/6 phosphorylation of RB. In the absence of CDK4/6 phosphorylation, RB remains bound to E2F1, thereby preventing E2F1 dependent transcription of S phase initiating functions. In contrast, the ARF gene product prevents MDM2-mediated polyubiquitination of p53, thereby inhibiting its proteasomal degradation. The resulting increase of p53 levels in the nucleus results in G1 arrest primarily through increased transcription of the CDK inhibitor p21CIP1.

Interestingly, both the INK4a-Rb and ARF-p53 pathways have also been implicated in the phenomenon of cellular senescence, wherein cultured primary cells exhibit progressive loss of proliferative capacity during passaging. This loss of proliferative potential occurs by activation of a differentiation-like program that causes stochastic G1 arrest, and is accompanied by characteristic morphological changes (reviewed in [4]). The senescence program is activated by a variety of signals, including progressive loss of telomeric sequences during repeated rounds of DNA replication, other forms of DNA damage, and the oxidative and nutritional deficiencies present under tissue culture conditions. It is likely that all of these intra- and extracellular stresses are present at various stages of tumorigenesis. In fact, similar underlying signaling events have been shown to regulate both cellular senescence and *in vivo* tumorigenesis. For example, cellular senescence can be triggered by overexpression of some oncogenes, including activated RAS. The induction of this has been shown to result in INK4a activation through the RAF/MEK/ERK kinase cascade-mediated activation of Ets family transcription factors [5]. Senescence has also been observed in preneoplastic lesions *in vivo*, as well as in tumors of patients treated with certain chemotherapeutic agents [6]–[9]. Thus, INK4a and ARF are key regulators of a cell-autonomous surveillance system that responds to oncogenic signals by triggering a program that results in exit from the cell cycle.

In addition, these pathways have been implicated as negative regulators of stem cell self-renewal capacity, suggesting a role for these proteins in maintaining a balance between suppression of tumor cell growth and promotion of self-renewal of healthy tissues [10]. For example, BMI1 is both a proto-oncogene and a key regulator of stem cell self-renewal capacity, and exerts much of this control through suppression of INK4a and ARF expression [11], [12].

Here we present an unbiased, genome-scale functional screen to identify regulators of INK4a. We report the identification of 14 proteins that robustly and specifically activate transcription through INK4a regulatory sequences, and demonstrate a role for one regulator, MEOX2, in the regulation of senescence through direct transcriptional activation of p16INK4a.

## Results and Discussion

### Functional screen for potential activators of INK4a

**Figure 1:**
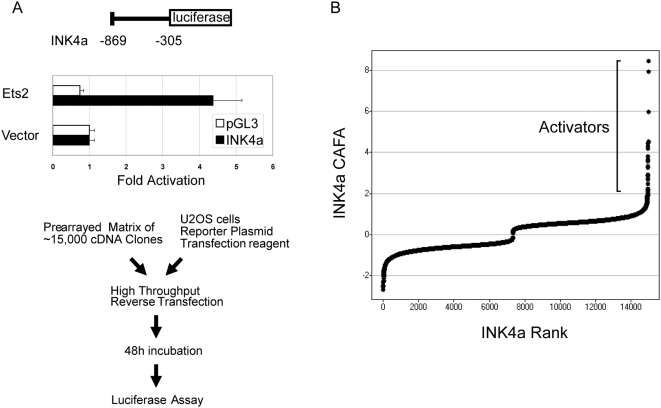
Transcriptional activators of INK4A identified by a functional genomics screening approach. (A) Screening strategy. Numbering indicates portion of the INK4a regulatory region, relative to the natural translational start site, inserted into the pGL3 luciferase reporter. Validation of the reporter was performed by cotransfection in U2OS cells under screening conditions. Mean luciferase values from four replicates are shown; error bars represent one standard deviation. (B) Screen results. Luciferase values from duplicate wells were normalized by plate, transformed and scaled to generate relative activity values (see Materials and Methods), then plotted according to rank.

We conducted a genome-wide functional screen using an arrayed cDNA library to identify transcriptional activators of INK4a. We first constructed a luciferase-based reporter harboring transcriptional regulatory sequences located upstream of the INK4a coding region (Fig. 1a, top) [5], [13]. This construct was validated by cotransfection with the known INK4a activator ETS2, which resulted in 4–5 fold activation of the reporter (Fig. 1a, middle) [5], [13]. We then employed a cDNA library consisting of approximately 15,000 arrayed full-length human cDNAs to identify activators of this reporter by transient cotransfection in U2OS osteosarcoma cells (Fig. 1a, bottom). Importantly, U2OS cells are deficient in INK4a expression due to methylation of the promoter sequences [14], and thus are resistant to induction of senescence by overexpression of cDNAs that activate transcription of INK4a. Analysis of duplicate genome-wide screens revealed 285 potential activators by the criterion of increasing luciferase expression by 2.5 fold or higher over each plate mean (Fig. 1b and Table S1). These include several known or anticipated activators of INK4a, such as JUNB and both ETS and RAS family members (ETS2, ERG and NRAS), confirming the utility of this approach (Table S1). Analysis of over-represented functional classes among these candidates using Gene Ontology (GO) analysis (GOstat (http://gostat.wehi.edu.au/)) revealed multiple transcription factors and related categories, encompassing over 50 of the activating cDNAs (P<0.05; Table S2). These potential INK4a-activating transcription factors include multiple members of several growth-regulatory signal transduction pathways as determined by the KEGG BRITE functional annotation (http://www.genome.jp/kegg/tool/search_pathway.html). These include MAPK and ErbB signaling, as well as associations with various cancers, consistent with the known regulation of INK4a transcriptional activity via pro-oncogenic signals (Table S3). The only significantly over-represented GO category unrelated to transcriptional regulation was receptor-mediated endocytosis (GO 0006898, P = 0.03; Table S2), which, we hypothesize, reflects the regulation of the INK4A locus by receptor-mediated extracellular signals.

**Figure 2:**
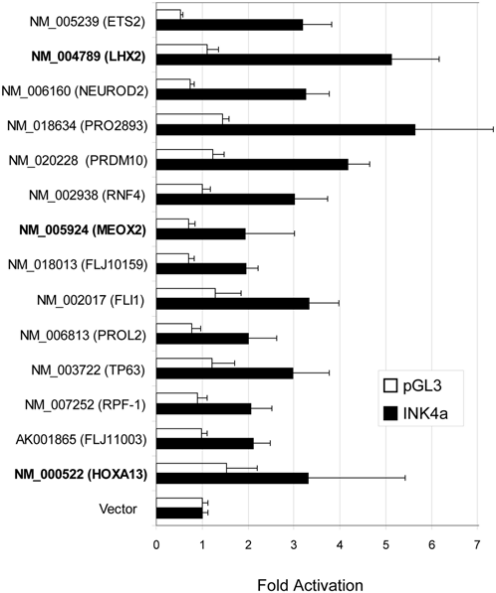
Confirmation of 14 INK4a activators. Results from four independent cotranfections of the indicated reporter with the indicated cDNA construct into U2OS cells are shown. Fold activation values were calculated for each experiment by normalizing luciferase levels of the indicated cDNA to the empty vector control (indicated as Vector). These values were then ranked by ratio of activation of the INK4a construct to activation of the empty luciferase reporter vector pGL3. Activators with relative activation values of two or more are shown. Homeodomain family members are in bold; error bars represent one standard deviation.

**Figure 3:**
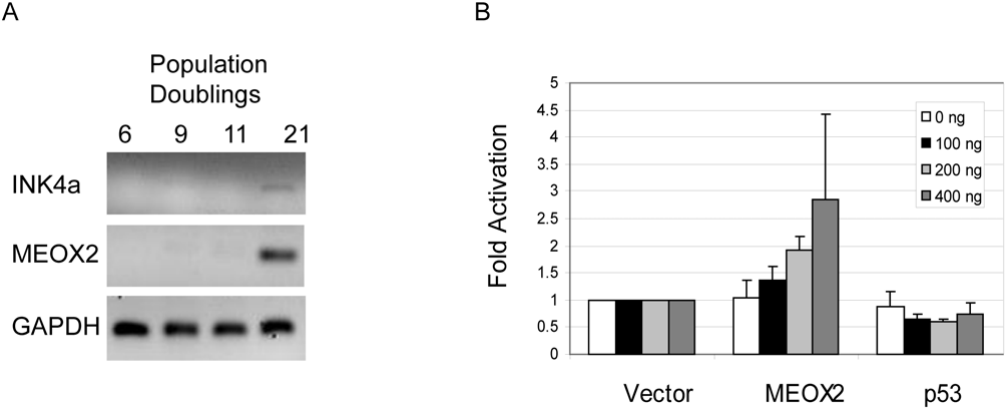
MEOX2 is a regulator of senescence in human cells. (A) MEOX2 is upregulated during senescence. Results of semiquantitative RT-PCR analysis on mRNA derived from primary human keratinocytes at the indicated number of population doublings are shown. (B) Dose-response for p16INK4a reporter activation by MEOX2. Results are shown from three independent cotranfections of U2OS with the indicated reporter and cDNA constructs as in Fig. 2. Error bars represent one standard deviation; p53 serves as a negative control.

To validate these potential INK4a regulators, a subset of the top activators was re-assayed in quadruplicate, and screened against the promoterless luciferase reporter backbone (pGL3) to eliminate nonspecific activators. Fourteen different cDNA clones showed both reproducible activation over the empty cDNA library vector control and at least two-fold activation of the INK4a reporter over the pGL3 backbone (Fig. 2). Several homeobox-containing transcription factors specifically activated INK4a in these assays (LHX2, HOXA13, and MEOX2; Fig. 2) as well as in another cDNA collection screened by the same protocol (HOXD8, HOXA11; J. Irelan & K. Quon, unpublished data). The homeobox superfamily is comprised of hundreds of transcription factors known to regulate pattern formation in early development, although roles for some members in adult tissues as well as in oncogenesis have been described [15]. To determine their potential roles in replicative senescence, we assessed the transcriptional regulation of the homeobox genes identified in the reporter screen by semiquantitative PCR during senescence in primary human cells. Of these, only MEOX2 mRNA levels were seen to increase concomitantly with INK4A during this process (Fig. 3a). Furthermore, we observed that MEOX2 can activate the INK4a reporter in a dosage-dependent manner (Fig. 3b). Taken together, these results suggest that MEOX2 plays a role in the regulation of INK4a transcription and cellular senescence.

### Forced expression of MEOX2 induces senescence in primary cells

**Figure 4:**
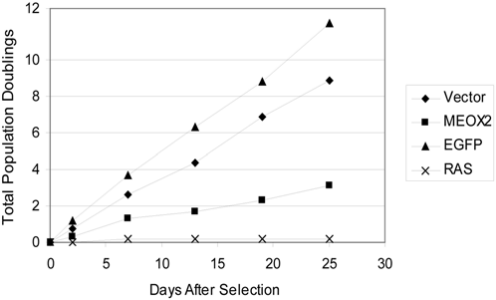
MEOX2 reduces proliferative capacity of primary human fibroblasts. Early passage WI38 fibroblasts were infected with retroviral particles expressing the indicated cDNAs, selected on puromycin, and assessed for proliferative capacity by periodic trypsinization and cell counting.

**Figure 5:**
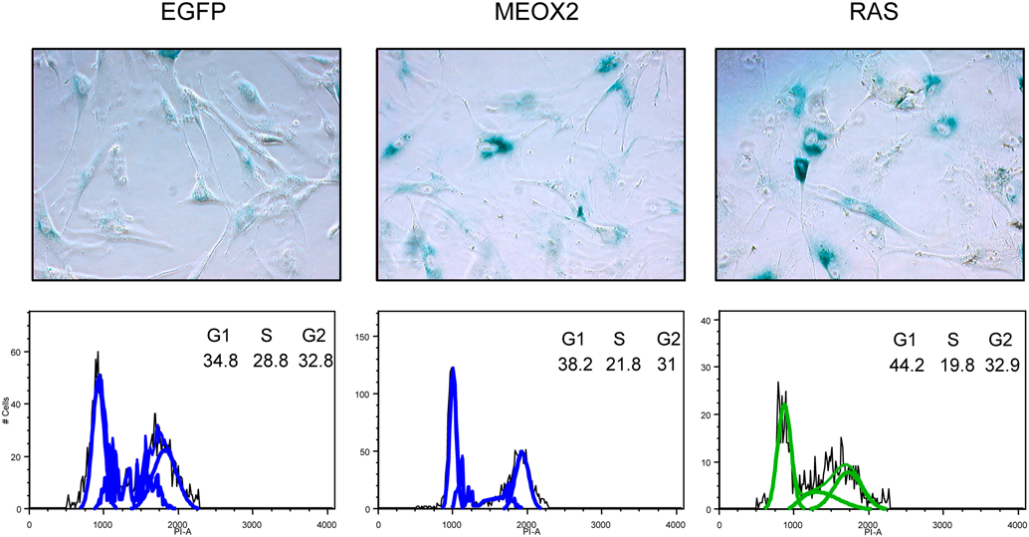
MEOX2 induces senescence phenotypes in primary human fibroblasts. Senescence-associated beta-galactosidase assays were performed on day 13 postselection. FACS analysis reveals accumulation of cells in the G1 phase of the cell cycle in response to MEOX2 expression.

MEOX2 (also known as GAX) overexpression has previously been shown to induce a p21CIP1 dependent cell-cycle arrest in vascular smooth muscle and vascular endothelial cells [16], [17]. This was demonstrated to occur through direct transcriptional activation of p21CIP1 by MEOX2 [18]. To determine whether MEOX2 can activate the senescence program in non-endothelial cell types, we introduced retroviral particles expressing MEOX2 in several primary human cell lines, including IMR90 lung fibroblasts, WI38 fibroblasts, and HEKn keratinocytes. Each of these cell types showed the hallmarks of accelerated senescence upon ectopic expression of MEOX2 (Fig. 4, Fig. 5, and unpublished data). Specifically, MEOX2 -expressing cells exhibited reduced proliferative capability and progressive cell size increase, relative to cells transduced with virus expressing enhanced green fluorescent protein (EGFP; Fig. 4 and unpublished data). Fibroblasts with forced MEOX2 expression also harbored significantly higher senescence-associated Beta-galactosidase activity, approaching the levels observed with activated RAS (Fig 5). Finally, MEOX2 expression also led to an accumulation of cells in the G1 phase of the cell cycle, and a concomitant decrease of cells in S phase, consistent with cells undergoing senescence (Fig. 5). Taken together, these results indicate that expression of MEOX2 can induce senescence in a variety of primary human cell types.

**Figure 6:**
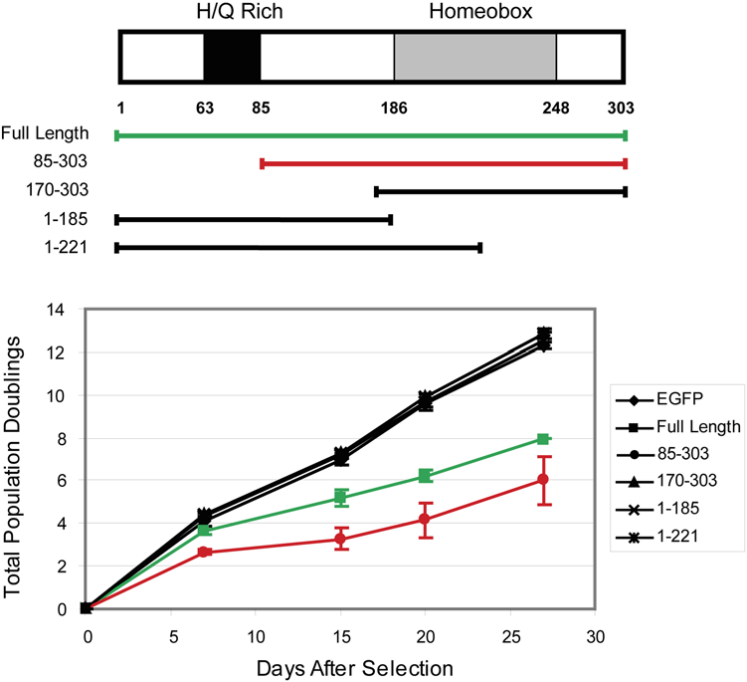
Structure/function analysis of the histidine/glutamine (H/Q) rich and homeobox domains of MEOX2. Retroviral constructs expressing the fragments of MEOX2 indicated by bars in the schematic (top panel) were assessed for induction of senescence as in Fig. 4. Results of three independent infections are depicted as mean values; error bars represent one standard deviation. Senescence-inducing constructs are shown in color.

To determine the structural requirements for senescence induction by MEOX2 overexpression, we introduced retroviral particles expressing different functional domains of MEOX2 into fibroblasts and subsequently assessed their effects on proliferative capacity. Deletion of the amino terminus, including the acidic histidine/glutamine rich domain in MEOX2, located between a.a. 63 and 85, did not affect the ability of the protein to induce senescence in fibroblasts; in fact this construct was even more active than the full-length construct (red vs. green line in Fig. 6), suggesting that this domain is dispensible for MEOX2 function. In contrast, none of the other constructs tested induced senescence. The homoeodomain of MEOX2 is located within the region corresponding to amino acids (a.a.) 186–248. Constructs lacking a complete homeodomain (a.a. 1–185, or 1–221) did not affect proliferation relative to the EGFP control, indicating that the DNA binding activity of MEOX2 is necessary for induction of senescence (black lines in Fig. 6). Similarly, a construct comprising only the homeodomain and C-terminal sequences (a.a.170–303) did not alter proliferative capacity, revealing that the homeodomain is not sufficient for senescence induction (Fig. 6). These data are consistent with a mechanism for MEOX2-driven induction of senescence that requires both DNA binding and transcriptional activation, mediated by sequences between a.a. 85 and 186, in addition to the homeodomain. Interestingly, it has been previously demonstrated that the histidine/glutamine domain is essential for the activation of p21CIP1 by MEOX2 in endothelial cells, suggesting an alternate mechanism of transcriptional activation of the INK4a promoter in fibroblasts [18]. We hypothesize that transcriptional regulation by MEOX2 may involve interactions with different coactivating proteins at the INK4a and p21CIP1 promoters, or with cell-type specific coregulators.

### MEOX2 activates INK4a expression to induce senescence

**Figure 7:**
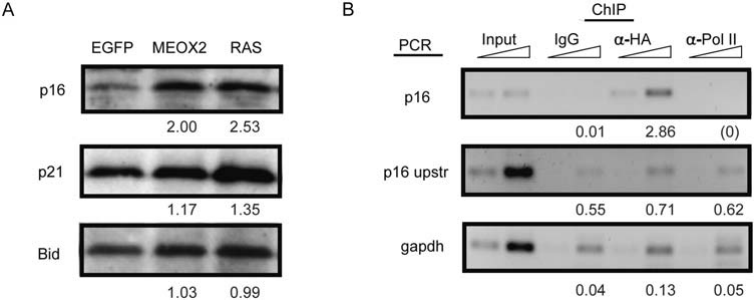
MEOX2 induces senescence through transcriptional activation of p16INK4a. (A) MEOX2 activates p16INK4a expression. Fibroblasts infected with retroviral constructs expressing the indicated cDNA (top) were harvested 9 days postinfection, and whole cell extracts assayed by western blot with the indicated antibodies (left). Bid serves as a loading control. Band intensities relative to the EGFP control are indicated. (B) MEOX2 binds to the p16INK4a promoter. Fibroblasts stably infected with a retroviral construct expressing hemagglutinin (HA)-tagged MEOX2 were harvested 15 days postinfection and subjected to chromatin immunoprecipitation with HA, PolII, or preimmune IgG antibodies. The resulting chromatin was assayed for enrichment of the indicated sequences by semiquantitative PCR with increasing cycle numbers. Band intensities relative to the input are indicated for the higher PCR cycle number; (0) indicates values at or below background intensity. The p16 primers amplified a fragment located 970 to 621 bp upstream of the INK4a ATG site, which is selectively enriched by MEOX2-HA immunoprecipitation. GAPDH and an amplicon located 5 kilobases upstream of the INK4a coding sequence (“p16 upstr”) serve as controls.

We next tested whether MEOX2 overexpression resulted in increased INK4a expression. Western blotting revealed that INK4a protein levels were increased two-fold by MEOX2 overexpression in fibroblasts, comparable to levels after expression of an activated form of RAS (Fig. 7a). p21CIP1 is the CDK inhibitor responsible for senescence induced by the ARF-p53 pathway, and has been implicated in MEOX2 overexpression-induced senescence in endothelial cells [16]. However, we observed only a modest increase in p21 levels in MEOX2-expressing fibroblasts (17%), suggesting that senescence induction likely occurs primarily through the INK4a-RB pathway (Fig 7a). To determine whether MEOX2 binds to the INK4a promoter directly, we conducted chromatin immunoprecipitation experiments on cells overexpressing HA-tagged MEOX2 (Fig 7b). Immunoprecipitation of MEOX2-HA strongly enriched for sequences located within the INK4a promoter region, but not control sequences located in GAPDH or 5 kb upstream of INK4a, indicating that MEOX2 binds to the INK4a promoter. Taken together, these results indicate that MEOX2 likely activates INK4a transcription by directly binding to the INK4a promoter.

**Figure 8:**
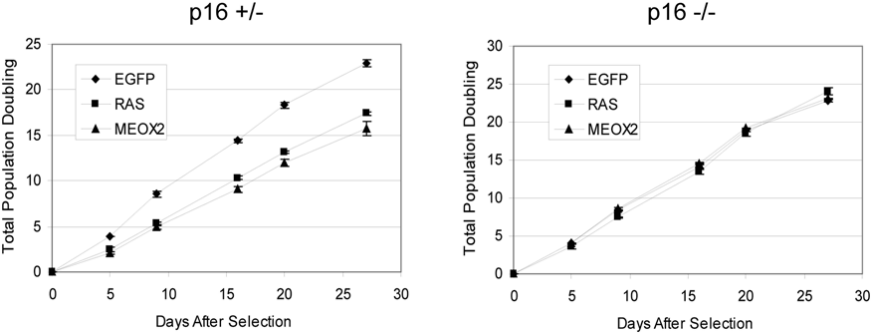
MEOX2-induced senescence requires p16INK4a. Early passage p16INK4a −/− (Leiden) or +/− (Foo3) fibroblasts were infected with retroviral particles and assayed for proliferation as in Figure 4. Mean values of three replicates are shown; error bars represent one standard deviation.

Finally, we tested whether MEOX2 induction of senescence in human fibroblasts requires p16INK4a. We utilized a pair of human diploid fibroblast cell lines isolated from individuals that are either heterozygous or homozygous mutant for an allele of CDKN2A that is linked to familial melanoma. This allele produces a nonfunctional form of p16INK4a and a product that retains functional ARF activity [19]. The ectopic expression of MEOX2 reduced proliferative capacity of the heterozygous control cells, but not the homozygous p16INK4a-deficient cells (Fig. 8), indicating that the activity of the INK4a pathway is required for MEOX2 mediated induction of senescence in human diploid fibroblasts.

Genome-scale functional screens have the potential to identify novel biological regulators, and to assign novel function to known regulators. Here we have identified a number of potential regulators of INK4a transcription, of which 14 were confirmed for activity and investigated further. One of these candidates was MEOX2, a diverged homeobox family member normally expressed in muscle precursor cells where it has a role in embryonic limb muscle development [20]. In adults, expression is largely limited to cardiovascular tissues. MEOX2 was originally identified as GAX (Growth Arrest Specific Homeobox), since it is upregulated during endothelial cell activation in an injury healing model, implicating a specialized and dynamic role for cell cycle regulation by this homeodomain protein in adult tissues [16]. In addition, MEOX2 has been suggested to play a role in the maintenance of normal brain vasculature in the context of Alzheimer's disease progression [21].

Here, we demonstrate a role for MEOX2 as a regulator of senescence in human keratinocytes and fibroblasts. Specifically, the transcription of this homeodomain protein is induced during the onset of cellular senescence in keratinocytes. Furthermore, we domonstrate that MEOX2 can directly activate INK4a transcription, resulting in the induction of senescence in fibroblasts. Interestingly, MEOX2 has been shown to also induce a cell cycle arrest in mouse endothelial cells in a p21CIP1-dependent manner. Structure-function analyses support distinct mechanisms for the regulation of p16INK4a and p21CIP1 in these cell types. Moreover, these differences may reflect the relatively greater importance of the ARF-p53-p21CIP1 axis over the INK4a-RB axis in senescence induction in mouse versus human cell types. Intriguingly, MEOX2 has recently been shown to be aberrantly methylated in non-small cell lung carcinoma, consistent with a tumor suppressor function for this homeodomain protein [22]. Taken together, these results suggest a regulatory role for MEOX2 in the INK4a-RB pathway for tumor surveillance in adult tissues.

## Materials and Methods

### Cell culture and analysis

Human diploid fibroblasts WI38, IMR90, Leiden, and Foo3 were grown on DMEM with 10% heat inactivated FCS and Pen/Strep/Glut (GIBCO #119665, 26170-43, 10378-16); Human neonatal keratinocytes (HEKn) were grown on Epilife or M154 supplemented with HKGS (Cascade Biologics C-001-5C, M-EPI-500-CA, M-154-500, and S100-5). Senescence-associated Beta-galactosidase activity was assayed with Cell Signaling staining kit (#9860). Cells were imaged at room temperature in PBS on a Nikon TE2000-U at 40× magnification, 0.13 N.A. Images were acquired with a Photometrics CoolSNAP HQ2, using Nikon ACT-1C software. FACS analyses were performed on ethanol-fixed cells stained with propidium iodide on a BD LSRII and the data were analyzed using FlowJo software.

### cDNA overexpression screen

U2OS osteosarcoma cells were reverse- transfected at a density of 4000 cells per well in 384-well plates that had been prespotted with 62 ng of Origene Trueclone library or control cDNA, along with 60 ng (pGL3-INK4a) reporter and a 3∶1 ratio of volume (ul) Fugene 6 transfection reagent (Roche #1 988 484) to total ug DNA as per manufacturer's instructions. After 48 h incubation, total luciferase activity was assessed by Bright-Glo (Promega #E2620). Raw luciferase values for each well were obtained (Acquest, LJL Biosystems) and normalized to their respective plate median values, then averaged to produce an average fold activation (“afa”) score. Each cDNA was ranked by afa score; the top 285 annotated activators are shown (Table S1). For reconfirmation, a set of 96 cDNAs chosen from among the top afa scores was re-assayed in quadruplicate, and raw luciferase values were normalized to empty vector controls.

### RT-PCR expression filter

mRNA from primary neonatal human keratinocytes (HEKn; Cascade Biologics) was isolated by RNeasy isolation kit (Qiagen #740104) and reverse transcribed into cDNA by the ThermoScript RT-PCR system (Invitrogen #11146-024) according to the manufacturers instructions. PCR was carried out using Platinum PCR supermix at 20, 25, and 30 cycles each consisting of 2 minute extension and 30 second 50 C annealing (gapdh) temperatures with the following primers: GAPDH – 5′-TCCCATCACCATCTTCCAG-3′ and 5′-ATGAGTCCTTCCACGATACC-3′; p16 (INK4a) - 5′-CAACGCACCGAATAGTACG and 5′-TTCTTTCAATCGGGGATGTC-3′; MEOX2 - 5′-GGAAAAGCGACAGCTCAGAC-3′ and 5′-AATTCCCGACAGCTCTGATG-3′.

### Chromatin Immunoprecipitation and Western Blotting

Chromatin Immunoprecipitations were carried out with the EZ ChIP (Upstate #17-371) kit using the manufacturer's instructions. Optimal chromatin shearing was obtained using 6 repetitions of 10 second sonications at 30% max on a Sonics Vibracell sonicator. For PCR amplifications, 2 ul of immunoprecipitated sample or 1 ul of input chromatin was incubated at 94 C for 5 minutes, followed by addition of 23 ul of Platinum PCR supermix (Invitrogen #10790-020) with each primer at 400 nM. 37 and 42 cycles of PCR using 2 minute extension and 30 second annealing at 48 C (p16, p16upstr) or 55 C (GAPDH). Primer sequences used for amplification are as follows: GAPDH - 5′-TCCCATCACCATCTTCCAG-3′ and 5′-ATGAGTCCTTCCACGATACC-3′; p16 – 5′-TACGACTAGAAAGTGTCCCCCTAC-3′ and 5′-TAGAACACTGAGCACTTTTTCTGG-3′; p16upst 5′-TAACCACAAGTGTGGCAAAAAG-3′ and 5′-GGAATAGAATTGCTGAGTCAAAGG-3′. The following antibodies were used for immunoprecipitaion and western blotting: p16INK4a (EMD biosciences #NA29), P21CIP1 (Santa Cruz Biotehnology # sc-56337), Bid (BD Biosciences #559681) and HA (Covance #MMS-101P). Results were scanned on a AlphaImager (Alpha Innotech), and mean pixel intensities in equivalent areas bounding each band were background corrected and quantified with ImageJ software (NIH).

## Supporting Information

Table S1 (0.09 MB XLS)

Table S2 (0.03 MB XLS)

Table S3 (0.03 MB XLS)
